# Acral palpable purpura in a patient with nephritis and shortness of breath

**DOI:** 10.1016/j.jdcr.2026.07.043

**Published:** 2026-07-30

**Authors:** Yasmine Gillespie, Alexander M. Hammond, Kiran Motaparthi

**Affiliations:** a University of Florida College of Medicine, Gainesville, Florida; b University of Florida Department of Dermatology, Gainesville, Florida

## Case description

**Fig 1:**
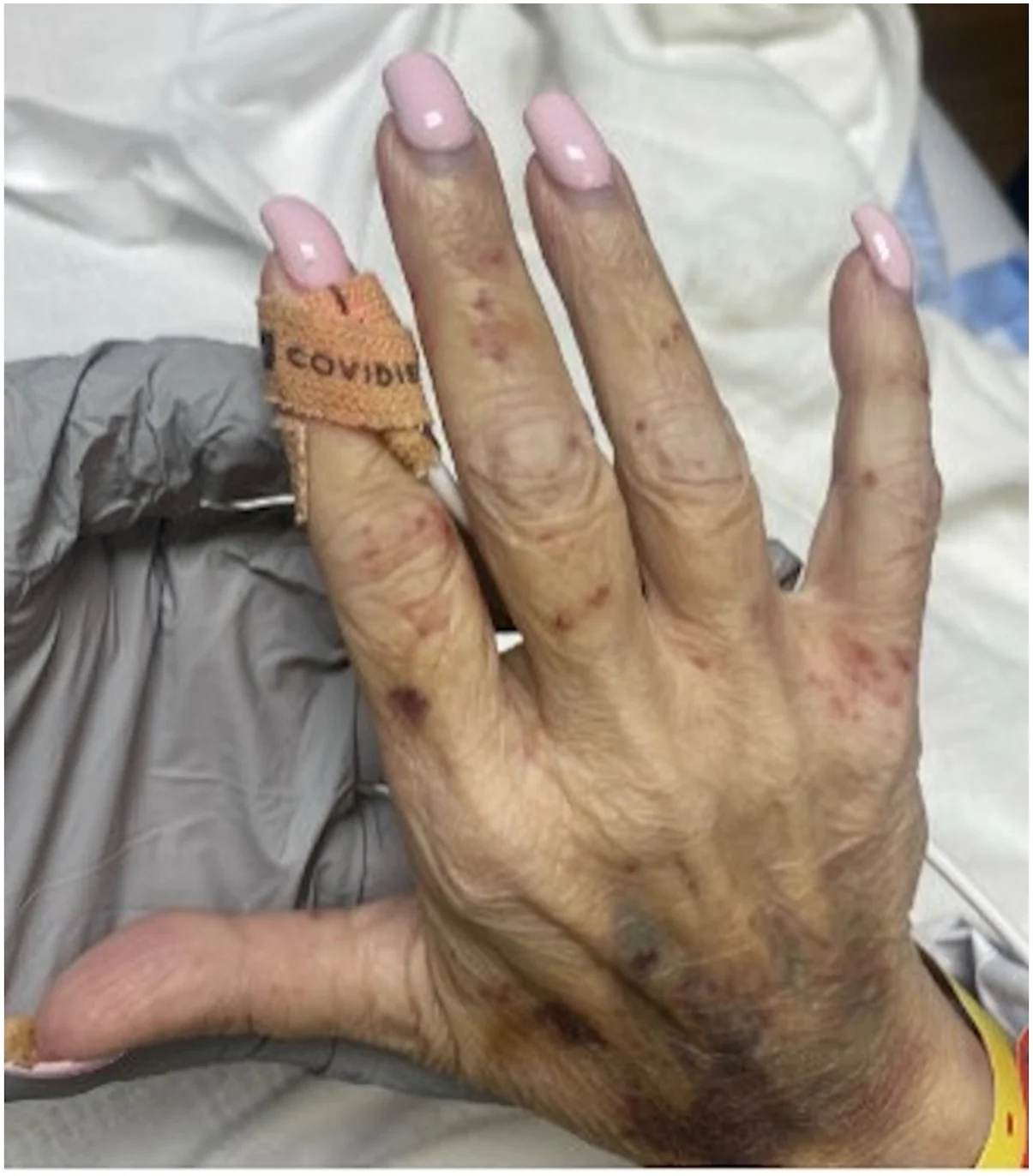
Palpable purpura on the digits.

**Fig 2:**
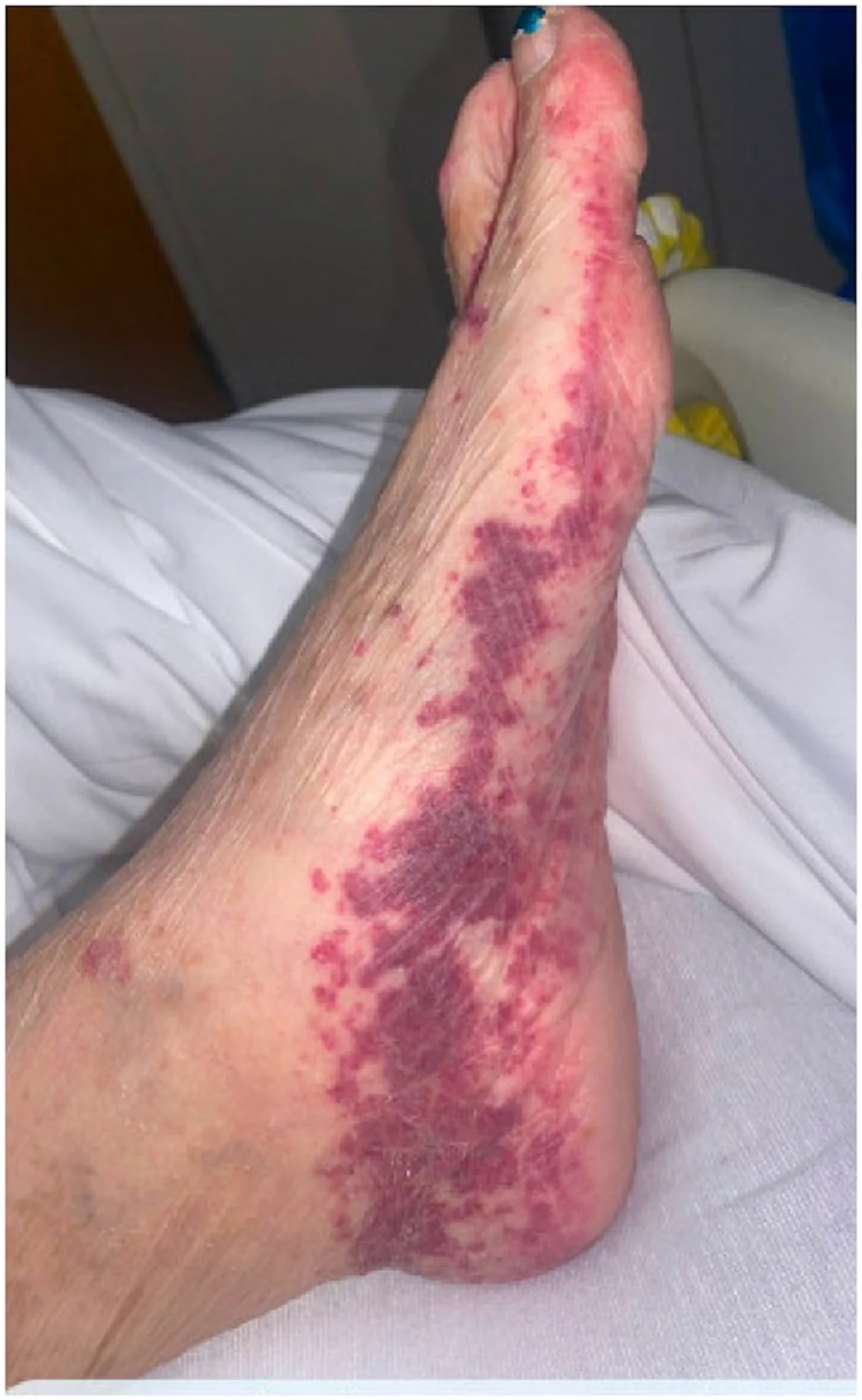
Palpable purpura and confluent purpuric patches on the medial and plantar foot.

A 60-year-old woman with stage III chronic kidney disease and hypertension on hydralazine for the past 3 years presented with a 1-day history of tender purpura on the bilateral lateral and plantar feet as well as her lateral digits (Figs 1 and 2). The patient also noted new-onset, worsening shortness of breath.

Urinalysis was notable for hematuria (20 red blood cells/HPF) and proteinuria (100 mg/dl), and comprehensive metabolic panel (CMP) revealed a creatinine of 2.18 mg/dL (baseline of 1.9 mg/dL). Chest computed tomography (CT) revealed a 2.2 cm cavitary nodule in the right lower lobe.

Punch biopsy demonstrated late findings of leukocytoclastic vasculitis, and direct immunofluorescence (DIF) revealed strong (3+) granular deposition of fibrinogen, IgM, and C3, without IgA deposition. Serologic testing showed a high-titer p-ANCA (>1:1280), dual PR3 (149 AU/mL) and MPO (21 AU/mL) positivity, positive rheumatoid factor, low complement (C3 and C4) levels, and concurrent ANA by indirect immunofluorescence (IFA) (>1:2560 with a homogeneous pattern), anti-dsDNA antibody by *Crithidia luciliae* IFA (1:20), and anti-histone antibody by ELISA (>7.0 units). Blood cultures and cryoglobulin testing were negative, and transesophageal echocardiography was unremarkable.

Due to persistent elevated creatinine, hematuria, and proteinuria, a renal biopsy was performed and demonstrated acute tubular injury and focal glomerulosclerosis without immune complex deposition, consistent with pauci-immune glomerulonephritis. The patient’s dyspnea and oxygen support requirement persisted.

**Question: What is the most likely diagnosis?**

**A.** IgA vasculitis
**B.** Cryoglobulinemic vasculitis
**C.** Drug-induced systemic lupus erythematosus
**D.** Hydralazine-induced ANCA-associated vasculitis
**E.** Septic vasculitis

**Correct answer:** **D.** Hydralazine-induced ANCA-associated vasculitis.

## Answer discussion

Hydralazine is a vasodilator used for hypertension and in rare cases can cause immune mediated adverse reactions, such as drug-induced systemic lupus erythematosus (SLE) and more rarely ANCA-associated vasculitis (AAV).^1^ On serology, patients commonly demonstrate elevated titers of MPO-ANCA, PR3-ANCA, and anti-histone antibodies.^2^ Additionally, ANA, anti-dsDNA antibodies, and hypocomplementemia are observed in 96%, 26%, and 44% of cases, respectively, all of which were present in this patient.^2^ Although anti-histone or ANCA autoantibodies may be positive in drug-induced SLE, anti-dsDNA antibodies are generally absent, making drug-induced SLE less likely.

Hydralazine-induced neutrophil injury triggers release of DNA, histones, and granule proteins that form neutrophil extracellular traps (NETs), which promote fibrinoid necrosis, amplify inflammation, and enhance MPO/PR3 antigen presentation, ultimately driving ANA, anti-histone, anti-dsDNA, and ANCA autoantibody production.^3^ The prolonged latency of several years following treatment initiation with hydralazine and the delayed manifestations of hydralazine-induced AAV reflect this pathophysiology.^2^

Combining positive serologies with acral palpable purpura raises suspicion for AAV. A detailed medication history and signs of systemic involvement can support the diagnosis of hydralazine-induced AAV. AAV in the kidneys classically demonstrates a pauci-immune glomerulonephritis pattern due to direct neutrophil-mediated injury,^4^ whereas DIF is positive in skin lesions in roughly 70% of cases, driven by classical complement pathway activation by IgG class ANCAs and protein deposition within cutaneous blood vessels.^5^ Although the patient’s creatinine elevation was modest and pulmonary disease was not specific to AAV, improvement in both was observed following discontinuation of hydralazine.

Prompt recognition of hydralazine-induced AAV is important: discontinuation of hydralazine is crucial, due to the risk of life-threatening renal and pulmonary disease.^3^ Systemic corticosteroids are the mainstay of therapy. Cyclophosphamide, rituximab, or plasma exchange are steroid-sparing treatments for refractory systemic disease.^6^

## Conflicts of interest

None disclosed.
